# Intraindividual Variability in Perceptual-Motor Performance Measured with Virtual Reality Among Military Veterans

**DOI:** 10.3390/brainsci16020185

**Published:** 2026-02-03

**Authors:** Scott L. Bruce, Michael Cooper, Carly Farmer, Audrey Folsom, Melanie Fulton, Jana Haskins, Cheryl Knight, Carlitta M. Moore, Johnathon A. Mullins, Amy Shollenbarger, Rashele Wade, Stacy Walz, Rebbecca Wellborn, Rachel Wilkins, Kendall Youngman

**Affiliations:** 1 Master of Athletic Training Program, Arkansas State University, Jonesboro, AR 72401, USA; 2 School of Nursing, Arkansas State University, Jonesboro, AR 72401, USA; mcooper@astate.edu; 3 Department of Medical Laboratory and Radiation Sciences, Arkansas State University, Jonesboro, AR 72401, USA; cafarmer@astate.edu (C.F.); askaggs@astate.edu (A.F.); kyoungman@astate.edu (K.Y.); 4 Department of Social Work, Arkansas State University, Jonesboro, AR 72401, USA; mfulton@astate.edu (M.F.); jhaskins@astate.edu (J.H.); cknight@astate.edu (C.K.); rwade@astate.edu (R.W.); rwellborn@astate.edu (R.W.); 5 Beck Center for Veterans, Arkansas State University, Jonesboro, AR 72401, USA; johnatho.mullins@smail.astate.edu; 6 Access and Institutional Engagement, Arkansas State University, Jonesboro, AR 72401, USA; camoore@astate.edu; 7 Department of Communication Disorders, Arkansas State University, Jonesboro, AR 72401, USA; ashollenbarger@astate.edu; 8 College of Nursing & Health Professions, Arkansas State University, Jonesboro, AR 72401, USA; swalz@astate.edu; 9 Department of Physical Therapy, Arkansas State University, Jonesboro, AR 72401, USA; rwilkins@astate.edu

**Keywords:** concussion, intra-individual variability, mental health, mild traumatic brain injury, perceptual latency time, response time, suicide ideation

## Abstract

**Background/Objectives:** Concussions produce a wide array of symptoms that are often subtle and difficult to quantify. One such symptom involves reaction or response time (RT), consisting of perceptual latency time (LT) and movement time (MT). This pilot study examined the relationship between concussion history, mental health, and perceptual-motor performance among military veterans using a virtual reality (VR)-based assessment. The primary outcome was intraindividual variability (IIV), defined as the standard deviation of an individual’s responses across repeated trials. **Methods:** Of 78 veterans who volunteered, 29 (22 males, 7 females) provided complete VR data. Participants completed surveys assessing concussion and combat history, mental health issues, and suicide ideation. During VR testing, participants responded to 40 trials requiring neck rotation, arm reach, and a step toward left or right virtual targets. Associations between predictors (e.g., concussion, mental health) and VR outcomes (RT, LT, IIV) were evaluated using Receiver Operating Characteristic (ROC) Area Under the Curve (AUC) values. **Results:** Concussion history was the strongest predictor of performance deficits. IIV metrics were sensitive indicators of both concussion and mental health issues. Univariable analyses yielded AUC values of 0.944–0.806 all of which were statistically significant (*p* ≤ 0.001), and multivariable analyses produced AUCs of 0.950–0.870 all of which were also statistically significant (*p* ≤ 0.001). Incongruent movements and longer LT values were especially discriminative. **Conclusions:** Veterans with concussion and mental health histories demonstrated quantifiable perceptual-motor impairments in VR environments. Findings support VR assessment as a feasible, sensitive tool for detecting subtle residual effects of concussion.

## 1. Introduction

A concussion occurs in a variety of ways. Most commonly, a blow to the head or the body causes injury. In the military, concussions can occur from a blow to the head or body, or by exposure to blasts, either directly or via pressure wave effects. The terms of mild traumatic brain injury (mTBI) and concussion are mostly interchangeable [1]. Of all head injuries occurring in the military, 80% are classified as mild [2]. Regardless of the cause, disturbance of the equilibrium of metabolic elements and compounds within the brain produces a variety of mostly subjective symptoms and potential impairments [1]. Some symptoms, like altered balance or gait, are observable; others manifest as temporary changes or deficits in memory or cognitive processing [3]. More subtle symptoms, such as impaired reaction time, can be difficult to quantify.

According to the World Health Organization (WHO), approximately one in seven people around the world live with a mental health disorder or issue. Mental illness disorders are defined as a “clinically significant disturbance in an individual’s cognition, emotional regulation, or behavior” [4]. The WHO outlines eight common mental health disorders: anxiety, depression, bipolar disorder, post-traumatic stress disorder (PTSD), schizophrenia, eating disorders, disruptive behavior and dissocial disorders, and neurodevelopmental disorders. Research demonstrates that individuals who have suffered concussions are at a greater risk for mental health issues, specifically depression, anxiety, PTSD and suicidality [5,6,7,8,9,10].

**Figure 1:**
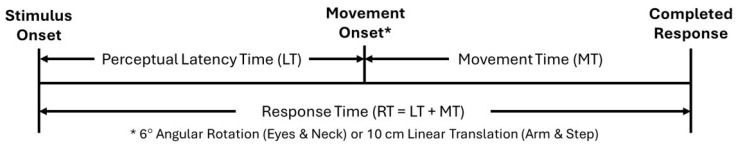
Operational definition of response time = perceptual latency time + movement time [14].

Reaction time or response time (RT) is a critical functional metric in athletics, in the military and with daily activities. RT includes perceptual latency time (LT)—the interval from appearance of the stimulus to movement initiation—and movement time—the duration from initiation to task completion [11,12] (Figure 1). Acute RT impairment is often apparent in the initial three days post-concussion and can persist for up to two months [13].

There are three types of RT: simple, choice, and composite/discrimination. Simple RT requires little advanced cognition. Procedural or choice RT calls for a go/no-go decision. Composite discrimination RT involves greater cognitive processing to distinguish between correct and incorrect responses [11,13].

Concussive injuries cause disruption to both brain chemicals and neural connectivity. Neuronal damage impedes impulse transmission, prompting the brain to reroute signals, similarly to detouring traffic, but often persisting at a slower transmission speed [15]. This damage can negatively affect many areas and networks of the brain, impacting attention, inhibition, and processing speed, which all influence RT. These longer RT are primarily due to extended LT, and are expressed with incongruent patterns or tests, but are amplified following a concussion [16,17].

There are two types of response inhibition: reactive (such as go/no-go choice tasks) and proactive (planned inhibition, for example, within the Eriksen flanker task) [18]. Unfortunately, conventional MRI and CT scan technologies are designed to identify macro-damage to the skull and brain, not the subtle microscopic neural or the neurometabolic disruptions that result from a concussion [19]. Advanced modalities such as diffusion Magnetic Resonance Imaging (dMRI), functional Magnetic Resonance Imaging (fMRI), Magnetic resonance spectroscopy (MRS) and arterial spin labeling all show promising clinical potential for identifying and characterizing the physiological and microstructural effects of concussion in research but are not readily available to the general medical community [20].

Being proficient in perceptual-motor skills requires interpreting environmental cues and producing coordinated motor actions [21]. These skills rely on perception (interpreting sensory data), motor execution (producing movement), and integration (combining sensory and motor responses at the right time and place) [22]. Routine perceptual-motor function can regress after concussion, heightening risks for affected individuals [23,24]. Recent studies have identified subtle impairments previously missed by conventional concussion assessment [16,25,26,27,28].

Virtual reality (VR) is a simulated environment in a real or imagine world created by a computer that allows the user to interact within that environment in which data is recorded regarding body movement in relation to the environment [29,30]. Immersive VR places the user into a three-dimensional simulated environment by using a head-mounted display removing one’s visual awareness of the real environment [14,30]. VR enables precision measurement of subtle impairment in a “gamified” environment. Traditional central tendency measures, such as those focusing on means, are insufficient for detecting individual changes in performance [31,32]. Intra-individual variability (IIV), defined as the standard deviation of performance across repeated trials, is a sensitive metric reflecting “the efficiency of executive control processes” [28] instead of random error or poor measurement reliability [28,31,32]. IIV encompasses discrepancy, dispersion, and inconsistency [28,33]. Discrepancy is the difference between the highest and the lowest score from a set of repeated measures. Dispersion refers to the inconsistency in scores over multiple test trials. Inconsistency is either the fluctuation from trial to trial in a single test session or from multiple test sessions conducted over the course of multiple days [28,32,33]

Most military personnel are young adults, sharing demographic similarities with collegiate and professional athletes [34]. Although sport-related concussion literature is extensive, military-specific data are limited. Similarities between concussions suffered within these two demographics exist with the primary difference being mechanism of injury [34,35]. Athletes and military personnel with a history of concussion are at a greater risk for mental health issues [5,6,7,9,36,37,38], with military personnel and veterans having the added burden of PTSD [5,9]. Similarities in the symptoms associated with persistent concussion symptoms and PTSD make the diagnosis between the two in military personnel and veterans challenging [35]. Suicide rates overall for veterans are 1.5 times higher than the general population. For both civilian and military populations, suicide rates by those with a history of suffering a concussion are two to three times higher compared to those without history of concussion [35]. Physical and psychological trauma to military personnel following an injury may produce complex changes to perceptual-motor function [2]. Much remains to be learned regarding reversibility and duration of deficits.

The aim of this study is to examine the relationship between concussion history, mental health, and perceptual-motor performance in veterans, measured via virtual reality. The primary outcome is intra-individual variability (IIV). The hypothesis is that veterans with a history of concussion and mental health issues will display worse IIV outcomes than their peers without such a history.

## 2. Materials and Methods

### 2.1. Study Design

This analysis is a subset of a larger cross-sectional research project by the Veterans’ Suicide Prevention Project (VSPP). The VSPP is an interprofessional group of allied health professors and researchers examining mental health issues and suicide ideation in military veterans. The protocol was reviewed and approved by the University’s Institutional Review Board.

### 2.2. Participants

Inclusion criteria were U.S. military veterans (≥18 years old) who were recruited via local veteran organizations and volunteered for the study. Exclusion criteria were non-veterans, younger than 18 years old, and inability to read English. No quotas or restrictions were applied beyond veteran status. Seventy-eight veterans participated, of whom 29 provided usable VR data for analysis.

### 2.3. Survey Instruments

All participants provided informed consent. Data were collected via demographic and health questionnaires. Concussion screening was based on self-report of previous concussion. No data were gathered regarding timing, mechanism, setting (military vs. non-military related occurrence or severity. Participants were instructed to respond “Yes” to this question, “if you have ever experienced a blow to the head that caused altered mental status, including any of the following: feeling confused, feeling dazed, feeling stunned, seeing stars, or felt like you got your bell rung.” Affirmative responses triggered further queries about the number of concussions [25,26,39]. Mental health was assessed via one-on-one interview with a social work mental health professional during which the General Anxiety Disorder (GAD-7) [40], and Patient Health Questionnaire (PHQ-9) were completed [41]. Veterans who scored ≥ 10 on the GAD-7 or the PHQ-9 were classified as having moderate anxiety or depression, respectively. Suicide ideation was identified by PHQ-9 question 9 [42]. Criteria for positive anxiety or depression cases were scores ≥ 10 on their respective instruments. Predictor variables included concussion history, multiple concussion history (≥2), depression, anxiety, and suicide ideation [5,41]. Post-traumatic stress disorder (PTSD) was examined as both a predictor and an outcome variable.

### 2.4. Virtual Reality

Testing was performed via VR headset (PICO Neo3 Pro Eye, PICO Immersive, Ltd., Mountain View, CA, USA). Calibration required participants to stand with arms abducted; targets were placed 30% beyond reach and outside peripheral vision, necessitating complex movements [27].

**Figure 2:**
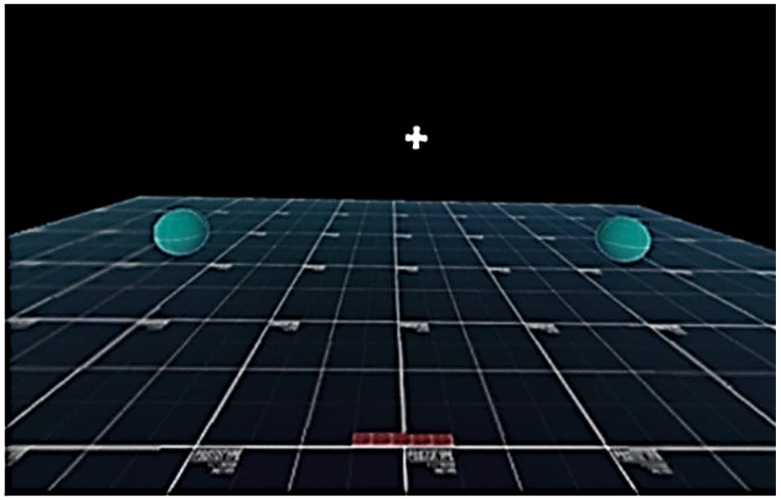
Virtual reality platform display. Green orbs are the virtual target located beyond peripheral field of vision from start position, require neck rotation to locate the correct response target (Figure reproduced with permission from Wilkerson, G. B., et al. (2023)) [14].

**Figure 3:**
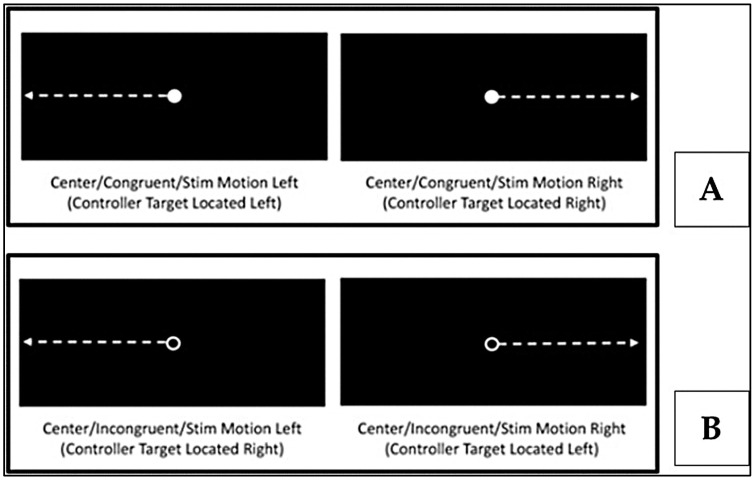
Example of visual cue combinations: congruent pattern ((**A**)—solid dot) and incongruent pattern ((**B**)—open ring) (Figure reproduced with permission from Wilkerson, G. B., et al. (2023)) [14].

Each VR session involved 40 visually cued trials requiring neck movement, arm reaching and whole-body step towards a virtual target in both the left and right directions (Figure 2). Cues differed for congruent (same direction as a solid dot [●]) (Figure 3A) and incongruent (opposite direction for an open ring [○]) trials, (Figure 3B) [14]. VR hardware recorded metrics such as mean RT and LT, and IIV across the six movement variables. Only correct responses were included in the analyses. Reliability was established with ICC values ranging from 0.837 to 0.922 [14].

### 2.5. Statistical Analysis

Descriptive data were calculated for height, weight, BMI, and time in service for males and females. Means and standard deviations were calculated for the number of correct and incorrect responses. Descriptive data for those veterans with both correct and incorrect overall responses are presented. Pair samples *t*-test were performed to compare the differences between the correct responses versus incorrect responses. Mean differences with 95% confidence intervals are also reported. Due to the small sample size, the Hedges’ *g* effect sizes are also reported [43]. Associations between predictors and VR outcomes were evaluated using the Receiver Operating Characteristic (ROC) Area Under the Curve (AUC) values [44,45]. Independent *t*-tests were calculated to examine for difference between the veterans who were positive for the predictor variable versus those who were not positive for the predictor variable. The *t*-ratio, mean differences with associated 95% confidence intervals and their associated *p*-values are all reported. For the purposes of this analysis, only ROC AUC metrics for the Top 10 and Top 25 results are reported. Higher RT, LT, and IIV values reflect neural inefficiency and are associated with concussion history [14,27]. Alpha level was set a priori at ≤0.05 for all statistical outcomes.

### 2.6. Use of Artificial Intelligence

This manuscript was authored by the research team and edited using generative AI (Perplexity AI v4.0.0, 2025, San Francisco, CA, USA). Perplexity was utilized strictly for grammar, clarity, and technical precision. All content was independently reviewed and approved by the authors to safeguard scientific integrity. The authors remain responsible for all content.

## 3. Results

Of the 78 veterans (62 males) who volunteered, 29 (22 males (79.9%), 7 females (24.1%)) provided complete VR data. Descriptive statistics are shown in Table 1 for both males and females.

Twenty of the 29 (68.97%) veterans assessed made the correct choice for all 40 trials (Table 2). Descriptive data for the correct and incorrect responses on the VR test are shown in Table 3. Paired *t*-tests were run to compare the correct, overall, average, to incorrect, overall, average for the neck, arms and steps response time, latency time and for the intraindividual variability (IIV) of each of those times. Only one pair of VR tests comparing correct versus incorrect results was statistically significant (Overall Average Arms LT, *t*[8] = −2.60, *p* = 0.032) (Table 4).

Twenty-eight predictor variables were analyzed against 72 VR outcome variables, producing a total of 2016 total variable combinations. Notable findings included the following:

- Concussion history and multiple concussion history were top predictors of impaired performance (Table 5 and Table 6).
- Most high-ranking predictors were related to mean neck and step RT; arm parameters were less frequently involved.
- IIV metrics emerged as prominent indicators of mental health issues and concussion history.
- All top AUCs were qualitatively described as either “Considerable” or “Excellent” [46] (Table 5, Table 6 and Table 7), and top Hedge’s *g* values were qualitatively described as “Large Effect” [47] (Table 5, Table 6 and Table 8).
- Independent *t*-test outcomes (Table 5 and Table 6) showed significant group differences for key variables.
- Multivariable analysis similarly highlighted concussion and mental health—depression, anxiety, PTSD, and suicide ideation—as major predictors (Table 5 and Table 6).
- Incongruent trials and LT values were especially discriminative.

Despite the small sample size these results are promising for further analyses with a greater number of participants.

## 4. Discussion

The purpose of this study was to examine whether those veterans with a history of concussion, involved in combat, and had been identified as having mental health issues affected their perceptual-motor performance as measured via virtual reality. The primary outcome measure was intraindividual variability (IIV). The hypothesis that veterans with a history of concussion and mental health issues will display worse IIV outcomes than their peers without such a history is accepted. This study indicates that veterans with a concussion history and mental health issues predicted impaired VR perceptual-motor performance and increased intraindividual variability.

Concussion history was the single strongest predictor; nearly all top associations involved previous history of concussion or multiple concussions. Like athletes, service personnel hide their concussions and their symptoms because they resist being separated from their peers or fear of letting their buddies down [49,50]. Mental health issues (particularly in combination with concussion history) were prominent. Combat exposure demonstrated less predictive specificity than anticipated, as consistent with the related literature [51,52].

Neck and step RT were stronger predictors than arm parameters. A possible explanation is that sufferers of concussion many times may involve cervical whiplash-like actions, but because of the severity of other concussion symptoms, cervical pain may be overlooked. If one does have cervical pain or cervical movement exacerbates other symptoms such as dizziness, headache or vestibular issues, they may subconsciously avoid moving their neck. After recovery from the concussion, this fear avoidance becomes engrained in the patient’s movement patterns. Slowly, they could be losing cervical ROM, or at least response time related to cervical movement [53,54].

The reasons behind step RT being a strong predictor are more difficult to quantify. Perhaps the veterans felt they could reach the virtual orb without stepping, then realizing after a few reps that it is easier to touch the orb if they moved their feet. Another plausible reason is that VR technology was new to most of the veterans, especially the older veterans. Having the headset placed on their head may have caused them to feel more unsteady, so they avoided moving their feet unless they felt they needed to move their feet.

Conventional central tendency statistics may not reveal subtle deficits post-concussion; however, Independent *t*-tests did provide statistically significant results for 23 of the Top 25 results for the univariable analysis and 15 of the Top 25 for the multi-variable analysis. Incongruent movement patterns were more discriminative in the multivariable analysis than in the univariable analysis, (19 of the Top 25 in the multivariable analysis, but only 9 of the Top 25 in the univariable analysis.) Longer LT values are likely due to slowing or altering of nerve signal propagation caused by the participant’s head injury history. Changes in one’s signal processing capabilities due to head trauma would lead to less consistent performance. IIV was a more precise marker of neural processing consistency [55,56,57]. IIV virtual reality variables accounted for all the Top 10 multi-variable combinations and 18 of the Top 25 overall. Neuronal rerouting leads to slower LTs, which in turn leads to slower RT [15,58,59]. VR provides a rigorous, quantitative assay and is supported by emerging studies [14,30,55,60]. However, these pilot study data indicate further analyses with a greater number and diverse population is needed to determine if these patterns are true or just by chance.

The interaction between concussion history, mental health issues and suicide ideation is a complicated process to pinpoint since every concussion is distinctively different. Issues faced by combat veterans are likely more significant and traumatic than those seen by the civilian population. Veteran suicide rates—especially in those with concussion history—remain high [61,62]. In the United States, 17.5 veterans commit suicide each day [62]. The need for more biopsychosocial focused research with veterans to explain these relationships is unmistakable.

### Limitations

Our study was not without limitations. As stated previously, only 29 participants provided usable VR data. This small sample limits our ability for generalization. Further, the small sample likely provided elevated Hedge’s *g* results. Because several of the variable combinations had a sensitivity of 1.0, this made 2 × 2 cross-tabulation analysis impossible without extrapolating the results. Sex-based analysis was not performed due to limited female representation (*n* = 7).

Another limitation was we did not ask about the context of the veterans’ concussion history (military vs. non-military) or more specific information about the severity or circumstances of their concussion history. Although VR generated >350 performance variables, our limited sample size constrained possible other analyses. Despite these issues, several key trends are evident which warrants further investigation with a larger, more diverse sample.

## 5. Conclusions

Veterans with concussion and mental health histories show quantifiable perceptual-motor deficits in VR environments, notably increased IIV and longer RT/LT values. These pilot data support the utility of immersive VR for detecting subtle deficits in brain function. Ongoing research should further explore tailored assessments and interventions. Additional research is needed with a larger, more diverse sample.

### Clinical Implications

Too often the clinician’s focus is on the macro-phenomena of cognitive and physical capabilities, and not on dual task concepts which are perceptual-motor performance. The overall purpose of this manuscript was to demonstrate that using the conventional neuropsychological, physiological models for assessing one’s readiness to return-to-activity is not giving clinicians a complete picture to the patient’s recovery from concussion. Further, this article attempts to draw attention to veterans’ history of concussion and how this history may relate to a veteran suffering from mental health issues. Secondarily, because this study was a pilot study, we hope we can inspire other researchers to further this work in perceptual-motor performance with military veterans in an effort to reduce the number of veteran suicides.

## Figures and Tables

**Table 1:** Demographic data of sample.

Mean ±SD	Males (*n* = 22)	Females (*n* = 7)
Height (cm)	172.72 ±14.81	178.49 ±6.93
Weight (kg)	100.37 ±19.45	72.69 ±15.69
BMI	25.06 ±7.99	31.44 ±5.31
Time in Service (years)	12.40 ±7.52	14.13 ±12.13

**Table 2:** Descriptive data for VR trails.

	Number Correct Targets	Number Incorrect Targets
Mean (±SD)	35.59 ±7.05	4.96 ±7.24
Maximum Number of Responses	40	20
Minimum Number of Responses	20	0

VR Test Consisted of 40 Trials.

**Table 3:** Descriptive data for correct and incorrect overall responses on the VR test.

		*n* ^a^	Mean	Standard Deviation
Pair 1	VR ^b^ Correct Overall Average Neck RT ^c^	9	1.43	0.392
	VR Incorrect Overall Average Neck RT		2.10	0.957
Pair 2	VR Correct Overall Average Arms RT	9	1.60	0.314
	VR Incorrect Overall Average Arms RT		2.12	0.618
Pair 3	VR Correct Overall Average Steps RT	9	1.71	0.386
	VR Incorrect Overall Average Steps RT		2.52	1.46
Pair 4	VR Correct Overall Average Neck LT ^d^	9	0.524	0.115
	VR Incorrect Overall Average Neck LT		0.541	0.305
Pair 5	VR Correct Overall Average Arms LT	9	0.477	0.166
	VR Incorrect Overall Average Arms LT		0.625	0.278
Pair 6	VR Correct Overall Average Steps LT	9	0.563	0.114
	VR Incorrect Overall Average Steps LT		0.581	0.264
Pair 7	VR Correct Overall IIV ^e^ Neck RT	7	0.324	0.182
	VR Incorrect Overall IIV Neck RT		0.451	0.554
Pair 8	VR Correct Overall IIV Arms RT	7	0.323	0.096
	VR Incorrect Overall IIV Arms RT		0.576	0.472
Pair 9	VR Correct Overall IIV Steps RT	7	0.344	0.136
	VR Incorrect Overall IIV Steps RT		1.19	1.04
Pair 10	VR Correct Overall IIV Neck LT	7	0.137	0.047
	VR Incorrect Overall IIV Neck LT		0.164	0.131
Pair 11	VR Correct Overall IIV Arms LT	6	0.196	0.070
	VR Incorrect Overall IIV Arms LT		0.222	0.201
Pair 12	VR Correct Overall IIV Steps LT	6	0.153	0.053
	VR Incorrect Overall IIV Steps LT		0.101	0.064

^a^ *n* = Number of veterans with both correct and incorrect responses for the variable. ^b^ VR = virtual reality. ^c^ RT = response time. ^d^ LT = latency time. ^e^ IIV = intraindividual variability.

**Table 4:** Results of paired *t*-test comparing correct responses to incorrect responses.

		Mean	Standard Deviation	95% CI ^a^ of the Difference	*t*	df ^b^	*p*-Value (2-Sided) ^c^	Hedges *g* Effect Size
Pair 1	VR Correct Overall Average Neck RT ^d^—VR Incorrect Overall Average Neck RT	−0.677	1.04	−1.478, 0.123	−1.95	8	0.087	1.15
Pair 2	VR Correct Overall Average Arms RT—VR Incorrect Overall Average Arms RT	−0.525	0.75	−1.10, 0.055	−2.09	8	0.070	0.836
Pair 3	VR Correct Overall Average Steps RT—VR Incorrect Overall Average Steps RT	−0.809	1.55	−2.00, 0.382	−1.57	8	0.156	1.72
Pair 4	VR Correct Overall Average Neck LT ^e^—VR Incorrect Overall Average Neck LT	−0.018	0.238	−0.201, 0.165	−0.22	8	0.829	0.264
Pair 5	VR Correct Overall Average Arms LT—VR Incorrect Overall Average Arms LT	−0.149	0.172	−0.281, −0.017	−2.60	8	**0.032**	0.190
Pair 6	VR Correct Overall Average Steps LT—VR Incorrect Overall Average Steps LT	−0.018	0.193	−0.167, 0.130	−0.29	8	0.783	0.214
Pair 7	VR Correct Overall IIV ^f^ Neck RT—VR Incorrect Overall IIV Neck RT	−0.127	0.608	−0.689, 0.436	−0.55	6	0.602	0.700
Pair 8	VR Correct Overall IIV Arms RT—VR Incorrect Overall IIV Arms RT	−0.253	0.470	−0.689, 0.182	−1.43	6	0.204	0.541
Pair 9	VR Correct Overall IIV Steps RT—VR Incorrect Overall IIV Steps RT	−0.848	1.04	−1.81, 0.112	−2.16	6	0.074	1.19
Pair 10	VR Correct Overall IIV Neck LT—VR Incorrect Overall IIV Neck LT	−0.027	0.111	−0.130, 0.075	−0.65	6	0.541	0.128
Pair 11	VR Correct Overall IIV Arms LT—VR Incorrect Overall IIV Arms LT	−0.026	0.208	−0.244, 0.192	−0.31	5	0.770	0.247
Pair 12	VR Correct Overall IIV Steps LT—VR Incorrect Overall IIV Steps LT	0.052	0.066	−0.017, 0.121	1.94	5	0.110	0.078

^a^ 95% Confidence interval for mean difference. ^b^ Degrees of freedom. ^c^ Two-sided *p*-value reported from paired-samples *t*-test (α ≤ 0.05). ^d^ Response time. ^e^ Latency time. ^f^ Intraindividual variability. Bolded *p*-values indicate the specific value is ≤0.05.

**Table 5:** Outcome of univariable analyses for area under the curve from the Receiver Operating Characteristic analysis for the association between the predictor variables and the VR variables along with the Independent *t*-test results.

Univariable Analyses									
Rank	Predictor Variable	Virtual Reality Variables	Area	*t*	df	Mean Difference	95% CI ^g^	Hedges *g*	*p*-Value ^h^
1	Multiple Concussion History (9/20) ^a^	Incongruent Average Neck RT ^c^ (=) ^d^	0.944	5.55	27.0	0.42	0.26, 0.57	2.18	**≤0.001**
2	Concussion History (19/10) ^a^	Incongruent Average Neck RT (≠) ^e^	0.937	5.13	26.2	0.35	0.21, 0.48	2.10	**≤0.001**
3	Concussion History	Overall Average Neck RT (=)	0.932	3.84	27.0	0.32	0.15, 0.49	2.05	**0.001**
4	Multiple Concussion History	Overall Average Neck RT (=)	0.928	4.56	27.0	0.37	0.20, 0.53	1.42	**≤0.001**
5	Multiple Concussion History	Overall Average Steps RT (=)	0.872	4.31	27.0	0.40	0.21, 0.59	1.56	**≤0.001**
6	Concussion History	Congruent Average Neck RT (≠)	0.858	3.64	26.3	0.30	0.13, 0.46	1.47	**0.001**
7	Multiple Concussion History	Overall IIV Steps RT (=)	0.856	4.06	27.0	0.43	0.21, 0.65	1.46	**≤0.001**
8	Concussion History	Overall Average Steps RT (=)	0.853	3.35	27.0	0.33	0.13, 0.53	1.44	**0.002**
9	Multiple Concussion History	Congruent Average Steps RT (=)	0.850	3.63	27.0	0.39	0.17, 0.61	1.42	**0.001**
10	Concussion History	Congruent Average Steps RT (=)	0.847	3.09	27.0	0.34	0.11, 0.56	1.41	**0.005**
11	Multiple Concussion History	Overall IIV Steps LT ^f^ (≠)	0.844	2.08	8.50	0.13	0.01, 0.27	1.30	0.069
12	Multiple Concussion History	Incongruent IIV Steps LT (=)	0.839	3.64	27.0	0.12	0.05, 0.19	1.36	**0.001**
13	Concussion History	Overall IIV Steps LT (=)	0.837	2.10	27.0	0.09	0.00, 0.19	1.35	**0.045**
14	Concussion History	Incongruent IIV Arms RT (≠)	0.832	3.97	24.2	0.19	0.09, 0.29	1.32	**0.001**
15	Multiple Concussion History	Incongruent Average Arms RT (=)	0.828	3.56	27.0	0.40	0.17, 0.63	1.30	**0.001**
16	Multiple Concussion History	Incongruent IIV Steps RT (=)	0.828	3.50	27.0	0.53	0.22, 0.83	1.30	**0.002**
17	Multiple Concussion History	Overall IIV Arms RT (=)	0.828	3.69	27.0	0.19	0.08, 0.29	1.30	**0.001**
18	Concussion History	Congruent IIV Arms RT (=)	0.821	1.60	27.0	0.10	0.03, 0.23	1.26	0.121
19	Concussion History	Overall IIV Arms RT (=)	0.821	2.25	27.0	0.27	0.02, 0.51	1.26	**0.033**
20	Concussion History	Overall IIV Neck LT (=)	0.821	1.99	27.0	0.09	0.00, 0.19	1.26	0.057
21	Suicide Ideation ^b^ (5/24) ^a^	Incongruent IIV Arms RT (=)	0.817	2.52	27.0	0.21	0.04, 0.37	1.24	**0.018**
22	Multiple Concussion History	Incongruent IIV Arms RT (≠)	0.811	3.01	9.70	0.23	0.06, 0.40	1.15	**0.014**
23	Multiple Concussion History	Incongruent IIV Neck LT (=)	0.811	3.23	27.0	0.10	0.04, 0.16	1.21	**0.003**
24	Multiple Concussion History	Overall IIV Arms LT (≠)	0.811	2.50	10.0	0.10	0.01, 0.18	1.15	**0.032**
25	Multiple Concussion History	Overall Average Arms RT (=)	0.806	3.23	27.0	0.36	0.13, 0.59	1.19	**0.003**

^a^ (Number of positive cases for the predictor variable/number of negative cases for the predictor variable. ^b^ Suicide ideation as indicated by positive response on PHQ-9, Question 9. ^c^ Response time. ^d^ (=), equal variances assumed. ^e^ (≠), equal variances not assumed. ^f^ Latency time. ^g^ 95% confidence interval for the mean difference. ^h^ Two-sided *p*-value reported from Independent *t*-test (α ≤ 0.05). Bolded *p*-values indicate the specific value is ≤0.05.

**Table 6:** Outcome of multivariable analyses for area under the curve from the Receiver Operating Characteristic analysis for the association between the predictor variables and the VR variables along with the Independent *t*-test results.

Multi-Variable Analysis									
Rank	Predictor Variable	Virtual Reality Variables	Area	*t*	df	Mean Difference	95% CI ^i^	Hedges *g*	*p*-Value ^j^
1	Both Multiple Concussion History and Depression (8/21) ^a^	Incongruent IIV ^d^ Steps LT ^e^ (=) ^f^	0.950	4.67	27.0	0.15	0.08, 0.21	2.26	**≤0.001**
2	Both Multiple Concussion History and Depression	Incongruent IIV Neck LT (=)	0.936	4.24	27.0	0.12	0.06, 0.18	2.09	**≤0.001**
3	Both Multiple Concussion History and Combat (4/25) ^a^	Incongruent IIV Neck LT (=)	0.935	3.82	27.0	0.15	0.07, 0.23	2.08	**0.001**
4	Both Multiple Concussion History and Combat	Incongruent IIV Steps LT (≠) ^g^	0.924	2.21	3.1	0.18	0.07, 0.44	1.50	0.110
5	Both Concussion History and Suicide Ideation ^b^ (4/25) ^a^	Incongruent IIV Arms RT ^h^ (=)	0.924	3.18	27.0	0.27	0.10, 0.45	1.97	**0.004**
6	Both Multiple Concussion History and Depression	Overall IIV Steps LT (≠)	0.921	2.26	7.3	0.15	0.01, 0.30	1.79	0.057
7	Both Multiple Concussion History and Suicide Ideation (3/26) ^a^	Incongruent IIV Arms RT (=)	0.917	3.04	27.0	0.30	0.10, 0.50	1.90	**0.005**
8	Both Multiple Concussion History and Suicide Ideation	Incongruent IIV Steps RT (=)	0.917	2.59	27.0	0.64	0.13, 1.14	1.90	**0.015**
9	Both Multiple Concussion History and Anxiety (4/25) ^a^	Overall IIV Steps LT (=)	0.913	1.25	27.0	0.08	0.05, 0.21	1.87	0.221
10	Either Combat or Depression (4/25) ^a^	Incongruent IIV Neck LT (=)	0.913	1.94	27.0	0.09	0.01, 0.18	1.87	0.063
11	Both Multiple Concussion History and Suicide Ideation	Overall IIV Steps RT (=)	0.903	2.17	27.0	0.41	0.02, 0.80	1.79	**0.039**
12	Either Combat or Anxiety (4/25) ^a^	Incongruent IIV Neck LT (=)	0.903	1.43	27.0	0.07	0.03, 0.16	1.79	0.165
13	Either Concussion History or PTSD ^c^ (6/23) ^a^	Overall IIV Neck RT (≠)	0.897	4.97	25.5	0.26	0.15, 0.36	1.74	**≤0.001**
14	Both Multiple Concussion History and Anxiety	Incongruent IIV Steps LT (=)	0.891	1.72	27.0	0.09	0.02, 0.20	1.69	0.097
15	Both Concussion History and Depression	Overall IIV Steps LT (=)	0.890	2.83	27.0	0.11	0.03, 0.20	1.69	**0.009**
16	Either Concussion History or PTSD	Incongruent Average Neck RT (=)	0.889	2.71	27.0	0.30	0.07, 0.53	1.68	**0.011**
17	Either Concussion History or PTSD	Incongruent IIV Steps RT (≠)	0.889	4.50	24.3	0.44	0.24, 0.64	1.67	**0.000**
18	Either Concussion History or Suicide Ideation (9/20) ^a^	Incongruent Average Neck RT (=)	0.882	3.52	27.0	0.32	0.13, 0.51	1.63	**0.002**
19	Either Concussion History or Suicide Ideation	Incongruent Average Steps RT (=)	0.882	1.57	27.0	0.27	0.08, 0.62	1.63	0.128
20	Both Multiple Concussion History and Depression	Incongruent Average Neck RT (=)	0.879	3.97	27.0	0.36	0.17, 0.55	1.61	**0.000**
21	Both Multiple Concussion History and Suicide Ideation	Incongruent Average Arms RT (=)	0.875	1.61	27.0	0.32	0.09, 0.72	1.58	0.119
22	Both Multiple Concussion History and Suicide Ideation	Incongruent IIV Steps LT (=)	0.875	1.45	27.0	0.09	0.04, 0.21	1.58	0.158
23	Both Multiple Concussion History and Suicide Ideation	Overall IIV Arms RT (=)	0.875	2.47	27.0	0.21	0.04, 0.38	1.58	**0.020**
24	Both Concussion History and Depression	Incongruent Average Neck RT (=)	0.874	3.58	27.0	0.30	0.13, 0.47	1.57	**0.001**
25	Either Combat or Depression	Incongruent Average Steps LT (=)	0.870	1.84	27.0	0.12	0.01, 0.25	1.55	0.077

^a^ (Number of positive cases for the predictor variable/number of negative cases for the predictor variable) first listing only. ^b^ Suicide ideation as indicated by positive response on PHQ-9, Question 9. ^c^ PTSD, post-traumatic stress disorder. ^d^ IIV, intraindividual variability. ^e^ Latency time. ^f^ (=), equal variances assumed. ^g^ (≠), equal variances not assumed. ^h^ Response time. ^i^ 95% confidence interval for the mean difference. ^j^ Two-sided *p*-value reported from Independent *t*-test (α ≤ 0.05). Bolded *p*-values indicate the specific value is ≤0.05.

**Table 7:** Qualitative descriptors for the strength of the association of the ROC AUC [46].

AUC Value	Interpretation
≥0.9	Excellent
0.8 to <0.9	Considerable
0.7 to <0.8	Fair
0.6 to <0.7	Poor
0.5 to <0.6	Fail

**Table 8:** Qualitative descriptors for the strength of the association of Hedge’s *g* effect size [48].

Effect Size	Strength of Association
0.20	Small Effect
0.50	Medium Effect
0.80	Large Effect

## Data Availability

The data presented in this study are available from the corresponding author upon institutional approval. The data are not publicly available, due to an institutional restriction on the release of data. A specific request from an individual who possesses research credentials must be reviewed and approved.

## Abbreviations

The following abbreviations are used in this manuscript:

PTSD	Post-traumatic stress disorder
RT	Reaction or RT
LT	Perceptual LT
MT	Movement time
VR	Virtual reality
IIV	Intraindividual variability
ROC	Receiver Operating Characteristic
AUC	Area Under the Curve
mTBI	Mild traumatic brain injury
dMRI	Diffusion Magnetic Resonance Imaging
fMRI	Functional Magnetic Resonance Imaging
MRS	Magnetic resonance spectroscopy
VSPP	Veterans’ Suicide Prevention Project
GAD-7	General Anxiety Disorder—7
PHQ-9	Patient Health Questionnaire—9
PHQ-9 Q9	Patient Health Questionnaire—9, Question 9
(=)	Equal variances assumed
(≠)	Equal variances not assumed
95% CI	95% Confidence Interval
